# Nocardiopsis codii sp. nov., and Rhodococcus chondri sp. nov., two novel actinomycetal species isolated from macroalgae collected in the northern Portuguese coast

**DOI:** 10.1099/ijsem.0.006483

**Published:** 2024-09-10

**Authors:** Mariana Girão, Zoé Lequint, Adriana Rego, Isabel Costa, Diogo Neves Proença, Paula V. Morais, Maria F. Carvalho

**Affiliations:** 1 CIIMAR - Interdisciplinary Centre of Marine and Environmental Research, University of Porto, Porto, Portugal; 2 ICBAS - School of Medicine and Biomedical Sciences, University of Porto, Porto, Portugal; 3 Polytech Clermont, University Clermont Auvergne, Clermont-Ferrand, France; 4 Department of Life Sciences, University of Coimbra, CEMMPRE, ARISE, Coimbra, Portugal

**Keywords:** actinomycetota, macroalgae, *Nocardiopsis codii*, Portugal, *Rhodococcus chondri*

## Abstract

Two novel actinomycetal strains, designated CC-R113^T^ and CC-R104^T^, were isolated from the tissues of two macroalgae collected on the northern Portuguese coast. Phylogenetic analyses based on the 16S rRNA gene showed that strain CT-R113^T^ belongs to the genus *Nocardiopsis*, being closely related to *Nocardiopsis umidischolae* 66/93^T^ and *Nocardiopsis tropica* VKM Ac-1457^T^, with 98.65 and 98.39 % sequence similarity, respectively. The clade formed between the three type strains was confirmed by phylogenomic analysis. The genome of strain CT-R113^T^ was 7.27 Mb in size with a G+C content of 71.3 mol %, with average nucleotide identity (ANI) values of 89.59 and 90.14 % with strains 66/93^T^ and VKM Ac-1457^T^, respectively. The major cellular fatty acids were identified as C_18 : 1_* ω*9*c*, iso-C_16 : 0_ and anteiso-C_17 : 0_. Menaquinone 10 (MK-10) was the major respiratory quinone. Comparative analysis of 16S rRNA gene sequences showed that strain CC-R104^T^ belongs to the genus *Rhodococcus* and is most closely related to *Rhodococcus pyridinivorans* DSM 44555^T^, with 98.24 % sequence similarity. However, phylogenomic analysis revealed that strain CC-R104^T^ establishes a clade with *Rhodococcus artemisae* DSM 45380^T^, being more distant from *Rhodococcus pyridinivorans* DSM 44555^T^. The genome of strain CC-R104^T^ was 5.34 Mb in size with a G+C content of 67.01 mol%. The ANI value between strains CC-R104^T^ and DSM 45380^T^ was 81.2 % and between strains CC-R104^T^ and DSM 44555^T^ was 81.5 %. The major cellular fatty acids were identified as C_18 : 1_* ω*9*c*, C_16 : 0_ and summed feature 3. Menaquinone 8 (MK-8) was the only respiratory quinone. For both CC-R113^T^ and CC-R104^T^, optimum growth was observed at pH 7.0, 28 °C and 0–5 % NaCl and whole-cell hydrolysates contained *meso*-diaminopimelic acid as the cell-wall diamino acid. On the basis of phenotypic, molecular and chemotaxonomic characteristics, strains CT-R113^T^ and CC-R104^T^ are considered to represent novel species, for which the names *Nocardiopsis codii* sp. nov. (type strain CT-R113^T^=LMG33234^T^=UCCCB172^T^) and *Rhodococcus chondri* sp. nov. (type strain CC-R104^T^=LMG33233^T^=UCCCB171^T^) are proposed.

## Introduction

The genus *Nocardiopsis,* proposed in 1976 [1], belongs to the family *Nocardiopsaceae* [2], order *Streptosporangiales* and class *Actinomycetes* [3], and currently comprises 50 taxa with validly published names (https://lpsn.dsmz.de/genus/nocardiopsis). Members affiliated to this genus are described as Gram-positive, aerobic, chemoorganotrophic, non-acid-fast, non-motile, and grow in filamentous forms with well-developed substrate mycelium and densely branched hyphae. Aerial mycelium can be sparse to abundant, fragmenting into spores of various lengths [4]. This genus is widely distributed in the environment, from deserts [5] to cold soils [6], deep ocean [7], coastal wetlands [8], and hypersaline to alkali soils [9]. An additional distinctive trait of *Nocardiopsis* is their ability to produce bioactive secondary metabolites with applications in several fields, including the pharmaceutical industry [10]. To date, 24 genomes of type strains of the genus *Nocardiopsis* have been sequenced with genome sizes ranging from 5.2 Mb (*Nocardiopsis alkaliphila* YIM 80379^T^ [11]) to 7.89 Mb (*Nocardiopsis umidischolae* 66/93^T^ [12]), and *in silico* G+C content between 67.5 (*Nocardiopsis alkaliphila* YIM 80379^T^ [11]) and 75.2 mol% (*Nocardiopsis trehalosi* [13]).

The genus *Rhodococcus*, redefined in 1977 by Goodfellow and Alderson [14], belongs to the family *Nocardiaceae*, order *Corynebacteriales* and class *Actinomycetes*, and currently comprises 55 taxa with validly published names (https://lpsn.dsmz.de/genus/rhodococcus). *Rhodococcus* species are metabolically fit to adapt and thrive in different ecological niches, booming in a wide range of environments, such as the ocean [15], terrestrial soil [16] and industrial and polluted sites [17,18], and living in association with animals [19] and plants [20], with some species being depicted as human pathogens [21]. Members of the genus *Rhodococcus* exhibit remarkable metabolic diversity and are particularly notable for their ability to biodegrade a variety of compounds. They act as catalysts for an array of biotransformations [22,24], in the production of biosurfactants [25] and as a source of bioflocculants [26]. In addition to these high-value industrial and ecological properties,* Rhodococcus* species have also been found to produce relevant antibiotics and anticancer compounds through their secondary metabolism [27,28], highlighting their pharmaceutical potential. Until now, 31 genomes of type strains of the genus *Rhodococcus* have been sequenced, with genome sizes ranging from 3.98 (*Rhodococcus corynebacterioides* NBRC 14404^T^) to 10.31 Mb (*Rhodococcus koreensis* DSM 44498^T^), and *in silico* G+C content between 61.7 (*Rhodococcus globerulus* NBRC 14531^T^) to 70.7 mol% (*Rhodococcus ruber* NBRC 15591^T^).

In this study, we describe two novel actinomycetal strains, designated CT-R113^T^ and CC-R104^T^. These strains were isolated from the tissues of two macroalgae, *Codium tomentosum* and *Chondrus crispus*, collected in the intertidal area of Mindelo beach, Portugal. According to a polyphasic approach based on chemotaxonomic, phenotypic and phylogenetic data, strains CT-R113^T^ and CC-R104^T^ represent a novel species within the genera *Nocardiopsis* and *Rhodococcus*, respectively.

## Isolation and ecology

Mindelo beach is located in Vila do Conde, northern Portugal (41.309298° −8.742228°). This area, like much of the northern Portuguese coastline, features numerous intertidal zones with rocky outcrops and tidal pools, providing habitats for various marine organisms, including macroalgae [29]. Intertidal zones are highly dynamic, with daily fluctuations of temperature, ultraviolet radiation, salinity and osmotic pressure, competition for space and resources, grazing pressures and impact of human activities [30,31]. Strains CT-R113^T^ and CC-R104^T^ were isolated from the tissues of two macroalgae specimens, *Codium tomentosum* and *Chondrus crispus*, respectively, collected in the intertidal rocky area of Mindelo beach, in early January 2020. The macroalgae were transported to the laboratory, washed with seawater to remove any attached particles, segmented in smaller pieces and macerated under sterile conditions, as described previously [32]. Briefly, the macerated tissues from the holdfast region (0.5 g) were placed in 2 ml tubes containing 1 ml sterile seawater and incubated in a water bath for 15 min at 58 °C. The resulting samples were ten-fold diluted until 10^−2^, and inoculated in selective culture media for actinomycetal isolation, supplemented with 1.5 % NaCl, cycloheximide (50 mg l^−1^), nalidixic acid (50 mg l^−1^) and nystatin (50 mg l^−1^), and kept at room temperature (± 24 °C). Strain CT-R113^T^ was purified on plates of actinomycete isolation agar and strain CC-R104^T^ on plates of nutrient-poor sediment extract agar and grown aerobically at 28 °C for 2 weeks. Stock cultures from both strains were maintained with 30 % (v/v) glycerol, at −80 °C [32].

## Phylogeny and phylogenomy

**Fig. 1.**
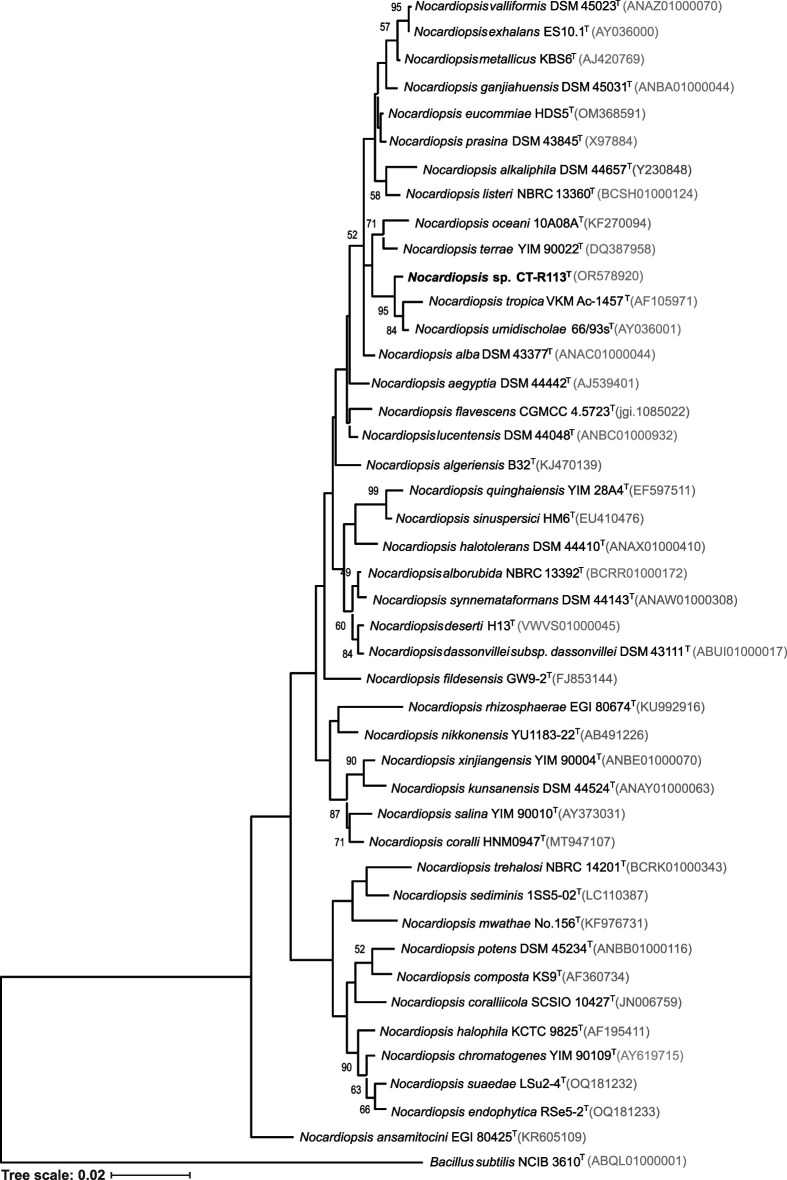
Maximum-likelihood phylogenetic tree based on 16S rRNA gene sequences (1551 nt), showing the relationship between strain CT-R113^T^ and the available type strains within the genus *Nocardiopsis*. Accession numbers are indicated in brackets. Values at the nodes indicate bootstrap values of 50 % and above, obtained based on 1000 resampling events. *Bacillus subtilis* NCIB 3610^T^ was used as an outgroup. Scale bar, 2 inferred nucleotide substitutions per 100 nucleotides.

**Fig. 2.**
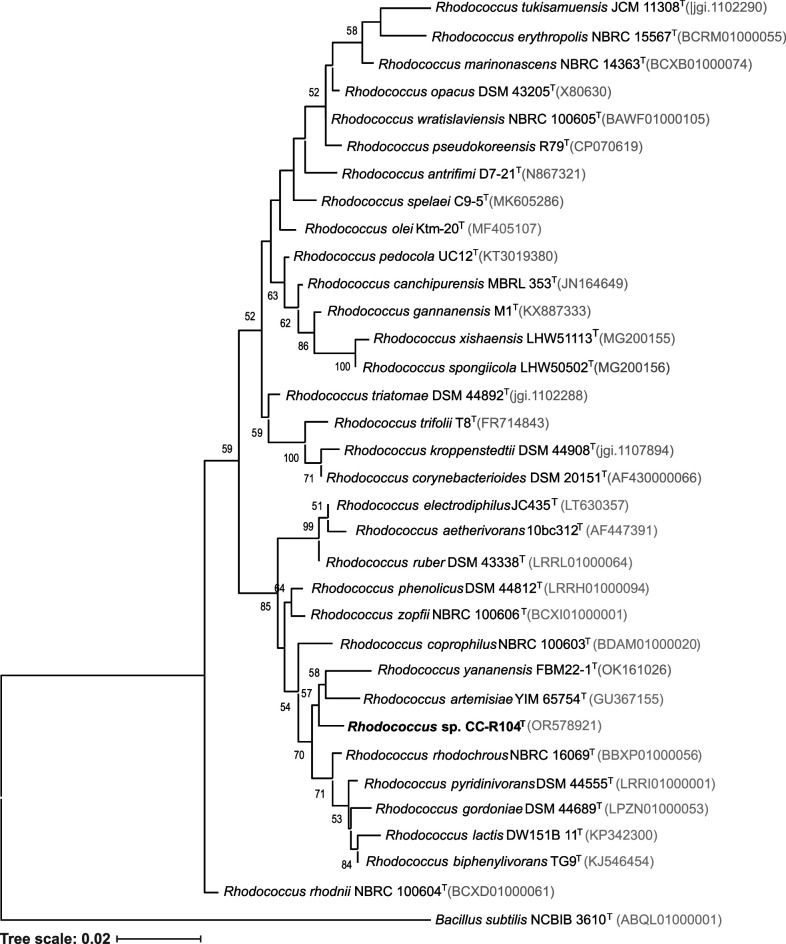
Maximum-likelihood phylogenetic tree based on 16S rRNA gene sequences (1553 nt), showing the relationship between strain CC-R104^T^ and the available type strains within the genus *Rhodococcus*. Accession numbers are indicated in brackets. Values at the nodes indicate bootstrap values of 50 % and above, obtained based on 1000 resampling events. *Bacillus subtilis* NCIB 3610^T^ was used as an outgroup. Scale bar, 2 inferred nucleotide substitutions per 100 nucleotides.

**Fig. 3.**
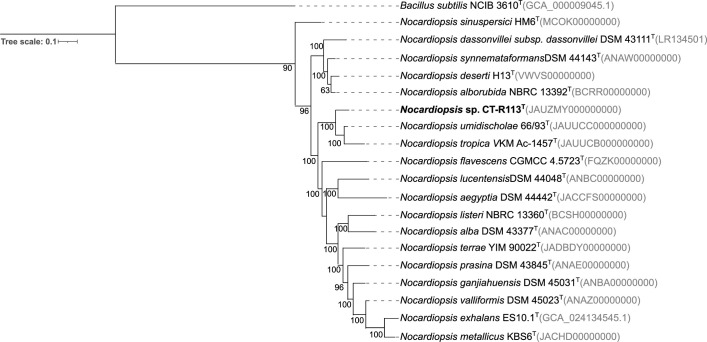
Maximum-likelihood phylogenomic tree based on 400 universal marker genes, showing the relationship between strain CT-R113^T^ and the closest related type strains within the genus *Nocardiopsis*. Accession numbers are indicated in brackets. Values at the nodes indicate bootstrap values of 50 % and above obtained based on 1000 resampling events. *Bacillus subtilis* NCIB 3610^T^ was used as an outgroup. Scale bar, 10 inferred nucleotide substitutions per 100 nucleotides.

**Fig. 4.**
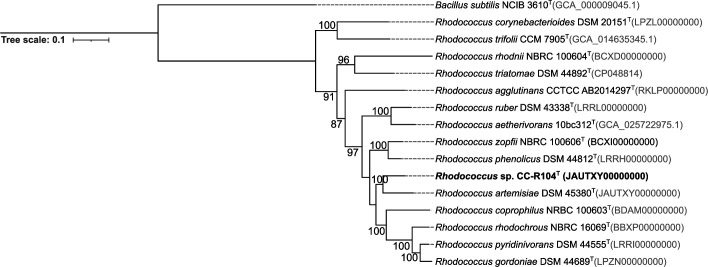
Maximum-likelihood phylogenomic tree based on 400 universal marker genes, showing the relationship between strain CC-R104^T^ and the closest related type strains within the genus *Rhodococcus*. Accession numbers are indicated in brackets. Values at the nodes indicate bootstrap values of 50 % and above obtained based on 1000 resampling events. *Bacillus subtilis* NCIB 3610^T^ was used as an outgroup. Scale bar, 10 inferred nucleotide substitutions per 100 nucleotides.

Phylogenetic relationships of both strains were inferred from 16S rRNA gene sequences. Extraction of genomic DNA, PCR amplification, and sequencing of 16S rRNA gene were carried out as described previously [33]. The 16S rRNA gene sequences of strains CT-R113^T^ and CC-R104^T^ were deposited in the NCBI with the accession numbers OR578920 and OR578921, respectively. As the original sequences had lengths <1400 bp, phylogenetic analyses were performed with the 16S rRNA gene sequences extracted from the sequenced genomes of CT-R113^T^ and CC-R104^T^. Pairwise similarity between each strain and their closely related type strains was calculated using the EzBiocloud database [34]. Phylogenetic trees were reconstructed using the maximum-likelihood (ML) and neighbour-joining (NJ) methods based on the 16S rRNA gene sequences of CT-R113^T^ and all the available type strains of the genus *Nocardiopsis* (Figs 1 and S1, available in the online Supplementary Material) and of CC-R104^T^ and all the available type strains of the genus *Rhodococcus* (Figs 2 and S2). Multiple sequence alignment was carried out using muscle [35] from the Geneious software package [36], and 1000 bootstraps were applied. mega-X [37] was used to build the tree and iTOL [38] to perform its final display and annotation. The highest 16S rRNA gene sequence similarity value for strain CT-R113^T^ was found with *Nocardiopsis umidischolae* 66/93^T^ (99.24 %) [12], followed by *Nocardiopsis tropica* VKM Ac-1457^T^ (99.24 %) [13]. Nonetheless, strain CT-R113^T^ formed an independent clade with these two type strains (Figs 1 and S1). The other closest hits were the strains *Nocardiopsis exhalans* ES10.1^T^ (98.88 %), *Nocardiopsis valliformis* DSM 45023^T^ (98.83 %), *Nocardiopsis metallicus* KBS6^T^ (98.76 %), *Nocardiopsis prasina* DSM 43845^T^ (98.69 %), *Nocardiopsis alba* DSM 43377^T^ (98.55 %), *Nocardiopsis aegyptia* DSM 44442^T^ (98.42 %), *Nocardiopsis lucentensis* DSM 44048^T^ (98.42 %), *Nocardiopsis terrae* YIM 90022^T^ (98.41 %), *Nocardiopsis ganjiahuensis* DSM 45031^T^ (98.21 %), *Nocardiopsis dassonvillei subsp. dassonvillei* DSM 43111^T^ (98.01 %), *Nocardiopsis deserti* H13^T^ (97.94 %), *Nocardiopsis listeri* NBRC 13360^T^ (97.90 %) and *Nocardiopsis alborubida* NBRC 13392^T^ (97.87 %). Interestingly, only *N. valliformis*, *N. aegyptia*, *N. lucentensis*, *N. terrae* and *N. ganjiahuensis* were retrieved from marine/saline environments, all from sediment samples [11,39,42]. The highest 16S rRNA gene sequence similarity value for strain CC-R104^T^ was found with *Rhodococcus pyridinivorans* DSM 44555^T^ (98.41 %) [43], followed by *Rhodococcus zopfii* NBRC 100606^T^ (98.13 %) [44]. However, results from the ML and NJ phylogenetic trees showed that strain CC-R104^T^ is most closely related to *Rhodococcus artemisiae* DSM 45380^T^, with a 16S rRNA gene sequence similarity of 97.71% (Figs 2 and S2). The other closest hits were strains *Rhodococcus rhodochrous* NBRC 16069^T^ (98.12 %), *Rhodococcus biphenylivorans* TG9^T^ (97.99 %), *Rhodococcus coprophilus* NBRC 100603^T^ (97.99 %), *Rhodococcus phenolicus* DSM 44812^T^ (97.99 %), *Rhodococcus yananensis* FBM22-1^T^ (97.80 %), *Rhodococcus artemisiae* YIM 65754^T^ (97.71 %), *Rhodococcus ruber* DSM 43338^T^ (97.70 %), *Rhodococcus gordoniae* DSM 44689^T^ (97.70 %), *Rhodococcus electrodiphilus* JC435^T^ (97.65 %), *Rhodococcus aetherivorans* 10bc312^T^ (97.50 %), *Rhodococcus lactis* DW151B^T^ (96.98 %), *Rhodococcus olei* Ktm-20^T^ (96.60 %), and *Rhodococcus gannanensis* M1^T^ (96.37 %). Remarkably, only *R. electrodiphilus* was retrieved from the marine environment [45]. The taxonomic evaluation of strains CT-R113^T^ and CC-R104^T^ was complemented with a phylogenomic study. Phylogenomic trees were computed using PhyloPhlAn 3.0 [46], based on 400 universal marker genes using each strain genome and the available genomes of the closest related type strains. mafft version 7 [47] was used to perform the multiple sequence alignment. ML trees using the LG substitution model were obtained. NJ trees were computed using mafft. Phylogenomic trees were imported and further edited using iTOL. FastANI [48] was used to calculate average nucleotide identity (ANI) and the Genome-to-Genome Distance Calculator [49] to calculate the digital DNA–DNA hybridization (dDDH) percentage between genomes. In line with the phylogenetic analysis, both phylogenomic trees exhibited the close relationships of CT-R113^T^ with *N. umidischolae* 66/93^T^ (ANI, 90.6 %; dDDH, 38.50 %) and *N. tropica* VKM Ac-1457^T^ (ANI, 89.9 %; dDDH, 40.90 %) (Figs 3 and S3). ANI values between strain CT-R113^T^ and the type strains included in the analysis were in the range of 90.6–82.4 % (Table S1), below the 95–96 % boundary that defines a new bacterial species. The dDDH values were all found to be below the 70 % threshold that defines a new species [50]. Phylogenomic data showed that strain CT-R113^T^ formed an independent branch within the genus *Nocardiopsis*. In agreement with the results based on the 16S rRNA gene sequences, both phylogenomic trees revealed a higher proximity between strain CC-R104^T^ and *R. artemisae* DSM 45380^T^ [51] (Figs 4 and S4). ANI values between strain CC-R104^T^ and the type strains included in the analysis were in the range of 81.7–77.9 % (Table S2), all below the previously mentioned threshold. The dDDH value between the CC-R104^T^ and DSM 45380^T^ genomes was 22.40 %. Even though the closest strain related to CC-R104^T^ based on ANI values is *Rhodococcus rhodochrous* NBRC 16069^T^ (ANI 81.7 %), the phylogenomic trees showed that strain CC-R104^T^ forms an independent branch with *R. artemisae* DSM 45380^T^ (ANI 81.2 %).

## Genome features

DNA extraction for whole genome sequencing of strains CT-R113^T^ and CC-R104^T^ was performed using the EZNA Bacterial DNA Kit (Omega Bio-Tek) according to the manufacturer’s instructions. Genomic DNA was sequenced using Illumina 2×250 bp paired-end technology and raw data were submitted to a bioinformatics pipeline (microbesNG). Briefly, identification of the closest reference genomes for reading mapping was done using Kraken 2 [52], read quality checks were performed using BWA-MEM [53], and *de novo* assembly was performed using SPAdes [54]. The genomes were annotated using the NCBI Prokaryotic Genome Annotation Pipeline and deposited at GenBank under the accession numbers JAUZMY000000000 and JAUZMZ000000000, respectively.

The genome of strain CT-R113^T^ was assembled into one contig with a length of 7 272 268 bp. dfast [55] results of completeness and contamination were 97.47 and 0.6 %, respectively. The *in silico* G+C content of strain CT-R113^T^ was 71.3 mol%, in line with the range for the genus *Nocardiopsis*. Annotation of the genome of strain CT-R113^T^ revealed the presence of 6518 coding sequences (CDS), 77 tRNA and three rRNA genes. As the genomes of *N. umidischolae* JCM 11758^T^ (=66/93^T^) and *N. tropica* JCM 10877^T^ (=VKM Ac-1457^T^) were not available, these strains were purchased from the Japan Collection of Microorganisms (JCM), their DNA extracted, and their genomes sequenced in order to be included in the analysis (GenBank accession numbers JAUUCC000000000 and JAUUCB000000000, respectively).

The genome of CC-R104^T^ was assembled into one contig of 5 341903 bp with 93.28 % completeness and 1.15 % contamination, according to dfast [55] analysis. The *in silico* G+C content of strain CC-R104^T^ was 67.01 mol%, in accordance with the typical values associated with the genus *Rhodococcus*. Annotation of the CC-R104^T^ genome revealed the presence of 4731 CDS, 56 tRNA and three rRNA genes. As the *R. artemisae* DSM 45380^T^ genome was not available, this strain was purchased from DSMZ (German Collection of Microorganisms and Cell Cultures – DSM45380), its DNA extracted, and its genome sequenced in order to be included in the analysis (GenBank accession number: JAUTXY000000000).

**Fig. 5.**
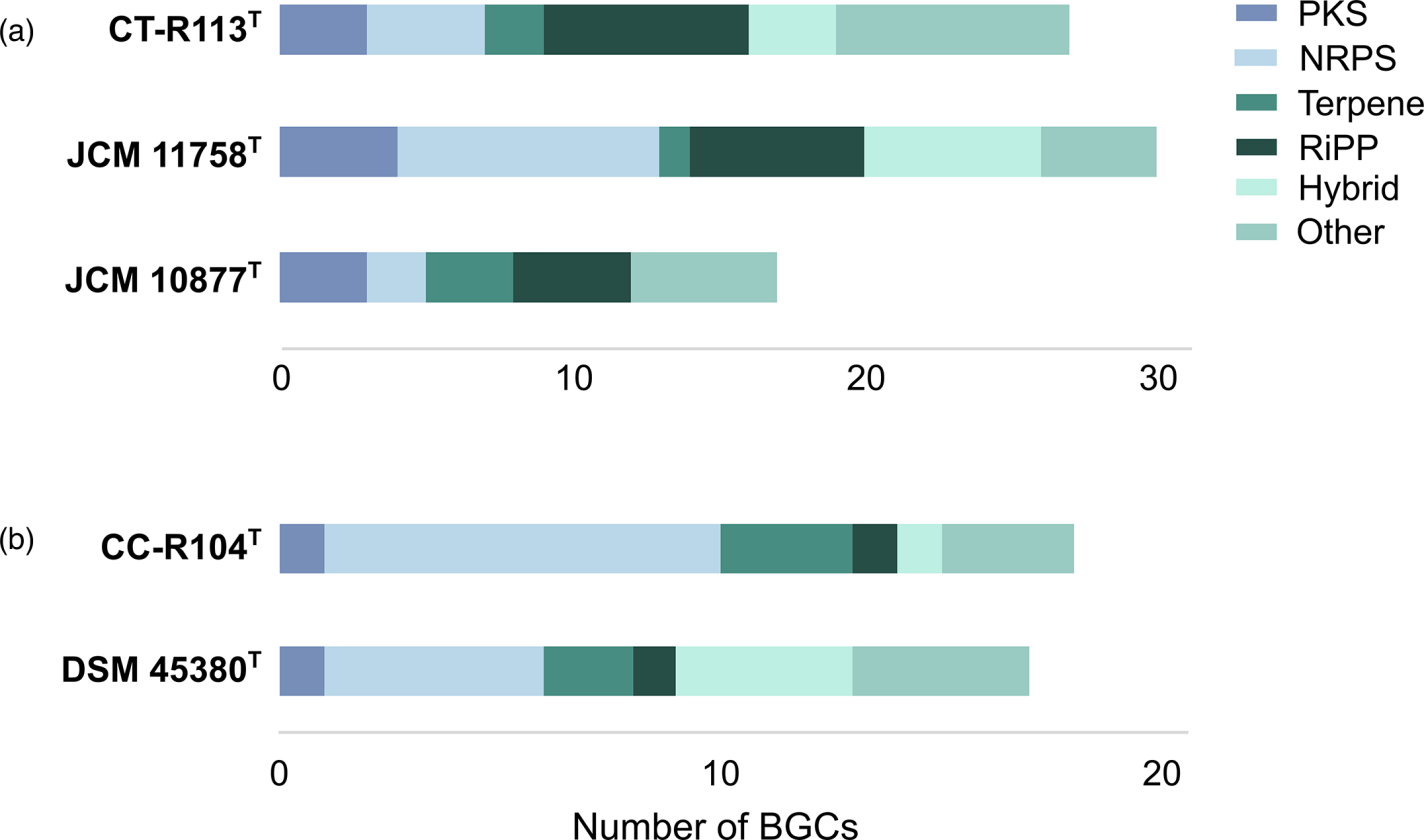
Distribution, number and type of biosynthetic gene clusters (BGCs) detected in (**a**) strain CT-R113^T^ and the two closest related type strains, JCM 11758^T^ and JCM 10877^T^, and (**b**) strain CC-R104^T^ and the closest related type strain DSM 45380^T^, as predicted by antiSMASH.

AntiSMASH [56] was used for the automated analysis of the genomes of strains CT-R113^T^ and CC-R104^T^, as well as of their closest related type strains, in order to identify and annotate gene clusters responsible for the biosynthesis of secondary metabolites, such as antibiotics and other bioactive compounds (Fig. 5a and b). Regarding strain CT-R113^T^ (and the closest type strains JCM 11758^T^ and JCM 10877^T^), the number and nature of the detected biosynthetic gene clusters (BGCs) in each genome was different, with strain JCM 11758^T^ being richer overall, closely followed by CT-R113^T^. There were distinctively fewer BGCs in strain JCM 10877^T^. Detailed *in silico* analysis of the genome of strain CT-R113^T^ revealed that most of the detected BGCs did not blast with known metabolites, or present low similarity values (< 40 %), highlighting the opportunity to uncover novel chemistry. The only hits recorded above the mentioned threshold were siderophore desferrioxamine E (100 % similarity, MiBiG accession BGC0001478), the non-ribosomal peptide metallophore coelibactin (90 % similarity, MiBiG accession BGC0000324), the terpene isorenieratene (87 % similarity, MiBiG accession BGC0001456) and the ribosomally synthesized and post-translationally modified peptide (RiPP) huimycin (70 % similarity, MiBIG accession BGC0002354). In comparison, only isorenieratene was confirmed in the genome of *N. umidischolae* JCM 11758^T^, showcasing the biosynthetic differences between the two species (Table S3). For strain CC-R104^T^ (and the closest type strain DSM 45380^T^), the analysis revealed differences in both the nature and number of the detected BGCs between each genome with strain CC-R104^T^ appearing to be particularly richer in terms of non-ribosomal peptide synthetase (NRPS) genes. Detailed *in silico* comparative analysis revealed that most of the detected BGCs in the CC-R104^T^ and DSM 45380^T^ genomes do not blast with known metabolites, or present low similarity scores (<40 %). The only exceptions recorded above the defined threshold for strain CC-R104^T^ were the terpenes 5-dimethylallylindole-3-acetonitrile from *Streptomyces coelicolor* A3(2) (55 % similarity, MiBiG accession BGC0002128) and isorenieratene from *Streptomyces griseus subsp. griseus* NBRC 13350 (42 % similarity, MiBiG accession BGC0000664). In comparison, only isorenieratene was found in the genome of DSM 45380^T^, together with the NAPAA (non-alpha poly-amino acids like e-polylysi) ε-poly-l-lysine biosynthetic gene cluster from the fungi *Epichloe festucae* (100 % similarity, MiBiG accession BGC0002174), showcasing the biosynthetic differences between the two species (Table S4).

Many of the detected BGCs likely codify for natural products with bioactive properties that are relevant for the strains' survival and thriving in the environment. For example, NRPs (non-ribosomal peptides) and RiPPs (post-translationally modified peptides), the two most abundant BGCs detected, can be responsible for the synthesis of molecules with antimicrobial, anti-fouling or antioxidant properties – traits that could benefit CT-R113^T^ and CC-R104^T^ living in this particular ecological niche.

## Physiology and chemotaxonomy

Unless otherwise stated, biomass for morphological, physiological and chemotaxonomic studies of strains CT-R113^T^ and CC-R104^T^ was obtained by cultivation on tryptic soy agar (TSA; MilliporeSigma), at 28 °C, for 2 weeks. Cultural characteristics were observed in a set of culture media, namely International *Streptomyces* Project (ISP) 2–ISP 7 [57], marine agar (MA; MilliporeSigma), potato–dextrose agar (PDA; HiMedia,), Czapek–dox agar (CZA; MilliporeSigma), TSA and glycerol–yeast–malt extract (GYM) agar [58]. The concentration of NaCl was adjusted to 1.5 % NaCl (w/v) in all culture media, with the exception of MA to which no additional salts were added. General morphological characteristics of the colonies were observed using a binocular magnifier. Physiological traits – temperature and pH range and tolerance to NaCl – were tested using TSA modified with 1.5 % NaCl (except in NaCl tolerance test), pH 7 (except in pH range test) at 28 °C (except in temperature range test), for 2 weeks. Growth was tested at 4, 12, 20, 28, 30, 32, 35, 37, 45 and 50 °C. A range of pH between 5.0 and 10.0 was tested, at intervals of 1.0 pH unit, using the following buffer system: acetate buffer (pH 5.0), phosphate buffer (pH 6.0–8.0), and Tris buffer (pH 9.0–10.0). Tolerance to NaCl concentrations was examined at 0, 1, 2, 3, 5, 10, 15 and 20 % (w/v). α-Cellulose, d-fructose, d-mannitol, raffinose, l-rhamnose, *myo*-inositol and sucrose were tested at 1 % (w/v) for carbon source utilization, as described previously [57]. Glycine, l-alanine, l-arginine, l-asparagine, l-proline, l-serine, l-threonine and l-tyrosine were tested at 0.1 % (w/v) for nitrogen source utilization, as described previously [59]. Enzymatic activity was tested using the API ZYM system (bioMérieux). Catalase activity was determined by using 3 % H_2_O_2_ (v/v), and gas production was identified as a positive reaction. Oxidase activity was evaluated using oxidase discs (Sigma-Aldrich) and blue coloration was identified as a positive reaction. For the analysis of lipoquinones, strains CT-R113^T^ (and the closest related strains JCM 10877^T^ and JCM 11758^T^) and CC-R104^T^ (and the closest related DSM 45380^T^) were cultured in tryptic soy broth (TSB), at 28 °C, for 48 h, at 150 r.p.m., harvested and lyophilized. Lipoquinones were extracted from freeze-dried cells, purified by TLC and separated by HPLC to identify the type present on the analysed strains [60]. Cells for fatty acid analysis were grown in TSB, at 28 °C, for 48 h, at 150 r.p.m. [61], and the fatty acid profiles were determined. Fatty acid methyl esters were obtained from fresh wet biomass and were separated, identified and quantified using the standard MIS Library Generation Software (Sherlock Microbial ID System, RTSBA 6 database, version 6.5) as previously described [62]. Analysis of isomers of 2,6-diaminopimelic acid (Dpm) and 2,6-diamino-3-hydroxypimelic acid was carried out by DSMZ Services (Leibniz-Institute DSMZ – Deutsche Sammlung von Mikroorganismen und Zellkulturen GmbH, Braunschweig, Germany) as described previously [63]. Gram staining was carried out according to the standard Gram stain method [64].

Strain CT-R113^T^ grew well on ISP 7, GYM, MA and TSA, and weakly on ISP 5 and CZA. No growth was observed on ISP 2, ISP 3, ISP 4 and PDA. This strain presented a yellowish substrate mycelium and a white branching aerial mycelium with white spores, when grown in the most proliferative culture medium, TSA (Fig. S5). Growth was observed at 12–28 °C, pH 6.0–10.0 and NaCl 0–10 % (w/v) on TSA, reaching its optimum at 28 °C, pH 7 and 0–5 % NaCl. Strain CT-R113^T^ was catalase positive and oxidase negative. This strain was found to grow on α-cellulose, l-rhamnose, *myo*-inositol and sucrose, with no visible growth on d-fructose, raffinose and d-mannitol. In contrast, both the closest type species, JCM 10877^T^ and JCM 11758^T^, grew well on d-mannitol, but presented no growth on l-rhamnose and *myo*-inositol. Regarding nitrogen sources, strain CT-R113^T^ was able to grow better on l-alanine, l-arginine, glycine and l-serine, with weak growth on l-asparagine, l-proline, l-threonine and l-tyrosine. Strains JCM 10877^T^ and JCM 11758^T^ were only able to grow in some of these substrates: the first strain did not show any growth with glycine and l-alanine and the second one with glycine and l-asparagine. The enzymatic profiling of CT-R113^T^ showed a positive reaction for alkaline phosphatase, esterase, leucine arylamidase and naphthol-AS-BI-phosphohydrolase and weak activity for esterase, lipase, α-glucosidase, β-glucosidase and α-fucosidase. Menaquinone 10 (MK-10) was the respiratory quinone of strain CT-R113^T^, as well as of its closest relatives JCM 10877^T^ and JCM 11758^T^. The major fatty acids (>10 %) of strain CT-R113^T^ were C_18 : 1_* ω*9*c*, anteiso-C_17 : 0_ and iso-C_16 : 0_, which accounted for 67.9 % of the total fatty acids (Table 1). The major three fatty acids of JCM 10877^T^ were also shared with strain CT-R113^T^ but with different relative percentages, which accounted for 57.2 % of the total fatty acids. Conversely, the third major fatty acid of JCM 11758^T^ was anteiso-C_15 : 0_, instead of iso-C_16 : 0_. The fatty acids anteiso-C_11 : 0_, C_17 : 0_ 3-OH and anteiso-C_17 : 1_* ω*9*c* were not detected in strain CT-R113^T^. Whole-cell hydrolysates contained *meso*-Dpm as the cell-wall diamino acid in strain CT-R113^T^, similar to strains JCM 11758^T^ and JCM 10877^T^. Comparative phenotypic, chemotaxonomic and genomic characteristics of strain CT-R113^T^ and of the two closest related type strains are presented in Table 2.

**Table 1.** Fatty acid compositions of strain CT-R113^T^, *Nocardiopsis tropica* JCM 10877^T^ and *Nocardiopsis umidischolae* JCM 11758^T^ Strains: 1, CT-R113^T^; 2, JCM 10877^T^; 3, JCM 11758^T^. All data are from this study. The major cellular fatty acids are in bold. tr, Trace amount (fatty acids amounting to <1 %); –, not detected or values lower than 0.45 %.

Fatty acid	1	2	3
C_8 : 0_ 3-OH	2.9±0.03	2.3±0.29	2.3±0.68
iso-C_10 : 0_	tr	tr	tr
anteiso-C_11 : 0_	–	–	tr
C_12 : 0_	1.1±0.01	tr	tr
anteiso-C_13 : 0_	tr	tr	tr
C_14 : 0_	tr	tr	tr
iso-C_14 : 0_	1.5±0	2.4±0.13	2.8±0.34
iso-C_15 : 0_	1±0.01	1.9±0.14	2.3±0.35
anteiso-C_15 : 0_	4.7±0.01	11.1±0.44	12.7±1.28
C_16 : 0_	1.9±0.04	2.2±0.11	2.1±0.29
iso-C_16 : 0_	**21.3±0.3**	**21.2±0.43**	**19.7±0.58**
C_17 : 0_	tr	1.5±0.13	1.2±0.18
iso-C_17 : 0_	3.3±0.04	3.8±0.12	3.9±0.12
anteiso-C_17 : 0_	**22.8±0.17**	**22.7±0.31**	**21.1±0.64**
C_17 : 0_ 3-OH	–	tr	tr
C_17 : 1_* ω*8*c*	2.3±0.02	2.4±0.09	1.8±0.09
anteiso-C_17 : 1_* ω*9*c*	–	–	tr
C_18 : 0_	3.9±0.15	4.9±0.3	5.6±0.81
C_18 : 0_ 10-methyl	1.0±0.04	tr	1.3±0.1
iso-C_18 : 0_	4.0±0.09	2.7±0.14	2.2±0.29
C_18 : 1_* ω*9*c*	**23.8±0.1**	**13.3±0.1**	**12.5±0.16**
anteiso-C_19 : 0_	tr	tr	tr
Summed feature 3*	1.5±0.05	2±0.16	2.3±0.28

†*Summed features are fatty acids that cannot be resolved reliably from another fatty acid using the chromatographic conditions chosen. The midi system groups these fatty acids as one feature with a single percentage of the total. Summed feature 3 contains C_16 : 1_* ω*7*c* and/or C_16 : 1_* ω*6*c*.

**Table 2.** Distinct phenotypic, chemotaxonomic and genomic characteristics of strain CT-R113^T^ and of its closest related type strains *Nocardiopsis tropica* JCM 10877^T^ and *Nocardiopsis umidischolae* JCM 11758^T^ Strains: 1, CT-R113^T^; 2, JCM 10877^T^; 3, JCM 11758^T^. +, Positive; −, negative. The predominant menaquinone of all strains is MK-10. The cell-wall diamino acid in all strains is *meso*-Dpm. All strains are catalase positive and oxidase negative. All strains produce aerial mycelium.

Characteristic	1	2	3
Isolation source	Macroalgae *Codium tomentosum*, Portugal	Rhizosphere of *Casuarina* sp., Seychelles	Indoor dust of a water-damaged school, Finland
Temperature range for growth (°C)	12–28	28–37*	10–37†
Optimum temperature for growth (°C)	28	28–30*	28
pH range for growth	6.0–10.0	6.0–9.0	7.0–10.0
Optimum pH for growth	7.0	7.0	7.0
NaCl range for growth (% w/v)	0–10	0–10*	0–7.5†
Optimum NaCl for growth (% w/v)	0–5	0–5*	0–5†
Carbon source utilization:			
α-Cellulose	+	−	+
d-Fructose	−	−	−
d-Mannitol	−	+	+
Raffinose	−	−	+
l-Rhamnose	+	−	−
*myo*-Inositol	+	−	−
Sucrose	+	+	+
Nitrogen source utilization:			
Glycine	+	−	−
l-Alanine	+	−	+
l-Arginine	+	+	+
l-Asparagine	+	+	−
l-Proline	+	+	+
l-Serine	+	+	+
l-Threonine	+	+	+
l-Tyrosine	+	+	+
Major fatty acids (>10 %)	C_18 : 1_* ω*9*c*, anteiso-C_17 : 0_ and iso-C_16 : 0_	anteiso-C_17 : 0_, iso-C_16 : 0_, C_18 : 1_* ω*9*c* and anteiso-C_15 : 0_	anteiso-C_17 : 0_, iso-C_16 : 0_, C_18 : 1_* ω*9*c* and anteiso-C_15 : 0_
Genome size (bp)	7 272 268	6 210 722	7 893 508
DNA G+C content (mol%)	71.3	72.3	72.7
BGCs (number and type)	27 (PKS, NRPS, terpene, RiPP, hybrid)	17 (PKS, NRPS, terpene, RiPP)	30 (PKS, NRPS, terpene, RiPP, hybrid)

*Data from [13].

† Data from [12].

Strain CC-R104^T^ grew well on MA, GYM and TSA, and weakly on ISP 2-ISP 7 and CZA. No growth was observed on PDA. This strain presented colonies with a dark yellow/orange coloration with no spores or aerial mycelium when grown in the most proliferative culture medium, TSA (Fig. S6). Growth was observed at 4–37 °C, pH 6.0–10.0 and NaCl 0–15 % (w/v), reaching its optimum at 28 °C, pH 7 and 0–5 % NaCl. Strain CC-R104^T^ was catalase positive and oxidase negative. This strain was able to use sucrose, *myo*-inositol, l-rhamnose, α-cellulose, d-fructose and raffinose as growth carbon sources, with no visible growth with d-mannitol. Conversely, the closest relative strain DSM 45380^T^, was able to use d-mannitol as a carbon source, but not α-cellulose. Regarding nitrogen sources, strain CC-R104^T^ was able to grow better on l-alanine, l-arginine, glycine, l-serine, l-asparagine and l-proline, with a weak growth on l-threonine and l-tyrosine. The same was observed for strain DSM 45380^T^. The enzymatic profiling of CC-R104^T^ showed a positive reaction for esterase, esterase lipase, leucine arylamidase and naphthol-AS-BI-phosphohydrolase, with weak activity for lipase, valine arylamidase and α-glucosidase. Menaquinone 8 (MK-8) was the only respiratory quinone of strain CC-R104^T^, in contrast with its closest relative DSM 45380^T^ for which MK-8 was not the exclusive quinone. The major fatty acids (>10 %) of strain CC-R104^T^ were C_18 : 1 _*ω*9*c*, C_16 : 0_ and summed feature 3, which accounted for 78.1 % of the total fatty acids (Table 3). The major three fatty acids of strain DSM 45380^T^ were also shared with strain CC-R104^T^ but with different relative percentages, which accounted for 85.8 % of the total fatty acids. Summed feature 9 was detected in DSM 45380^T^ but not in strain CC-R104^T^. Only strain CC-R104^T^ possessed C_15 : 1 _*ω*6*c*, C_17 : 0_, C_17 : 1 _*ω*8*c* and summed feature 6. Whole-cell hydrolysates contained *meso*-Dpm as the cell-wall diamino acid in strain CC-R104^T^, similar to strain DSM 45380^T^. Comparative phenotypic, chemotaxonomic and genomic characteristics of strain CC-R104^T^ and of its closest related type strain DSM 45380^T^ are presented in Table 4.

**Table 3.** Fatty acid composition of strain CC-R104^T^ and *Rhodococcus artemisiae* DSM 45380^T^ Strains: 1, CC-R104^T^; 2, DSM 45380^T^. All data is from this study. The major cellular fatty acids are in bold. tr, trace amount (fatty acids amounting to <1 %); –, not detected or values lower than 0.45 %.

Fatty acid	1	2
C_14 : 0_	2.3±0.2	2.7±0.1
C_15 : 1_* ω*6*c*	tr	–
C_16 : 0_	**22.7±0.8**	**32.6±0.4**
C_17 : 0_	3.6±0.3	–
C_17 : 1_* ω*8*c*	7.8±0.9	–
C_18 : 0_	3.5±0.4	2.2±0.1
C_18 : 0_ 10-methyl	tr	6.6±0.3
C_18 : 1_* ω*9*c*	**33.0±3.6**	**21.5±0.5**
Summed features:*		
**3**	**22.4±1.7**	**31.7±0.3**
6	tr	–
9	–	1.4±0.1

†*Summed features are fatty acids that cannot be resolved reliably from another fatty acid using the chromatographic conditions chosen. The midi system groups these fatty acids as one feature with a single percentage of the total. Summed feature 3 contains C_16 : 1_* ω*7*c* and/or C_16 : 1_* ω*6*c*. Summed feature 6 contains C_19 : 1_* ω*9*c* and/or C_19 : 1_* ω*11*c*. Summed feature 9 contains C_16 : 0_ 10-methyl and/or iso-C_17 : 1_* ω*9*c*.

**Table 4.** Distinct phenotypic, chemotaxonomic and genomic characteristics of strain CC-R104^T^ and of its closest related type strain *Rhodococcus artemisae* DSM 45380^T^ Strains: 1, CC-R104^T^; 2, DSM 45380^T^. +, Positive; −, negative. The predominant menaquinone of both strains is MK-8(H_2_). The cell-wall diamino acid in both strains is *meso*-Dpm. Both strains are catalase positive and oxidase negative. Neither of the strains produces aerial mycelium.

Characteristic	1	2
Isolation source	Macroalgae *Chondrus crispus*, Portugal	Plant *Artemisia annua* L.,China
Temperature range for growth (°C)	4–37	10–40*
Optimum temperature for growth (°C)	28	20–37*
pH range for growth	6.0–10.0	6.0–9.0*
Optimum pH for growth	7.0	7.0–8.0*
NaCl (% w/v) range for growth	0–15	0–7*
Optimum NaCl for growth (% w/v)	0–5	0–7*
Carbon source utilization:		
α-Cellulose	+	−
d-Fructose	+	+
d-Mannitol	−	+
Raffinose	+	+
l-Rhamnose	+	+
*myo*-Inositol	+	+
Sucrose	+	+
Nitrogen source utilization:		
Glycine	+	+
l-Alanine	+	+
l-Arginine	+	+
l-Asparagine	+	+
l-Proline	+	+
l-Serine	+	+
l-Threonine	+	+
l-Tyrosine	+	+
Major fatty acids (>10 %)	C_18 : 1_* ω*9c*,* C_16 : 0_, C_16 : 1_* ω*7*c* and/or C_16 : 1_* ω*6*c*	C_16 : 1_* ω*7*c* and/or C_16 : 1_* ω*6*c*, C_16 : 0_ and C_18 : 1_* ω*9*c*
Genome size (bp)	5 341 903	7 088 132
DNA G+C content (mol%)	67.01	66.2
BGCs (number and type)	18-PKS, NRPS, Terpene, RiPP, Hybrid	17-PKS, NRPS, terpene, RiPP, hybrid

*Data from [51].

## Description of *Nocardiopsis codii* sp. nov.

*Nocardiopsis codii* (co'di.i. N.L. gen. n. *codii*, of the algal genus *Codium*).

Gram-positive, aerobic, halotolerant, spore-forming, catalase-positive and oxidase-negative actinomycetota. Colonies on TSA, modified with 1.5 % NaCl, are small, opaque, circular with regular edges with a light-yellow coloration. In all the proliferative culture media (ISP 7, ISP 5, CZA, GYM, TSA and MA, all supplemented with 1.5 % NaCl, except MA), a dense mycelium with white spores is produced. No growth is observed on ISP 2–ISP 4 and PDA. Growth occurs at pH 6.0–10.0 (optimum, pH 7.0), at 12–28 °C (optimum, 28 °C) and with 0–10 % (w/v) NaCl (optimum, 0–5 %). Sucrose, *myo*-inositol, l-rhamnose and α-cellulose can be used as sole carbon sources, but not d-fructose, raffinose and d-mannitol. l-Alanine, l-arginine, glycine and l-serine can be used as sole nitrogen sources, but growth is weaker when using l-asparagine, l-proline, l-threonine and l-tyrosine. Menaquinone 10 (MK-10) is the only respiratory quinone. The major fatty acids are C_18 : 1_* ω*9*c*, anteiso-C_17 : 0_ and iso-C_16:0._ Whole-cell hydrolysates contain *meso*-Dpm as the cell-wall diamino acid. The genome size of strain CT-R113^T^ is 7 272268 bp and the G+C content of the genomic DNA is 71.3 mol%. The NCBI GenBank accession number for the genome assembly is JAUZMY000000000 and the accession number of the 16S rRNA gene sequence is OR578920. The type strain, CT-R113^T^ (=LMG 33234^T^=UCCCB 172^T^), was isolated from the tissues of the macroalgae *Codium tomentosum* collected on the northern Portuguese coast.

## Description of *Rhodococcus chondri* sp. nov.

*Rhodococcus chondri* (chon'dri. N.L. gen. n. *chondri*, of the algal genus *Chondrus*).

Gram-positive, aerobic, halotolerant, non-spore-forming actinomycetota. Colonies on TSA, modified with 1.5 % NaCl, are small, opaque, smooth and circular with regular edges presenting a dark yellow/orange coloration which intensity increases over time. No spores or vegetative mycelium are formed. Cells grow well on MA, GYM and TSA culture media and weakly on ISP 2–ISP 7 and CZA, all modified with 1.5 % NaCl except MA. Growth occurs at pH 6.0–10.0 (optimum, pH 7.0), at 4–37 °C (optimum, 28 °C) and with 0–15 % (w/v) NaCl (optimum, 0–5 %). Test for catalase activity is positive and for oxidase activity is negative. Sucrose, *myo*-inositol, l-Rhamnose, α-cellulose, d-fructose and raffinose can be used as sole carbon sources, but not d-mannitol. l-Alanine, l-arginine, glycine, l-serine, l-asparagine and l-proline can be used as sole nitrogen sources, but growth is weaker with l-threonine and l-tyrosine. Menaquinone 8 (MK-8) is the only respiratory quinone. The major fatty acids are C18 : 1 *ω*9*c*, C16 : 0 and summed feature 3. Whole-cell hydrolysates contained *meso*-Dpm as the cell-wall diamino acid. The genome size of strain CC-R104^T^ is 5 341903 bp and the G+C content of the genomic DNA is 67.01 mol%. The NCBI GenBank accession number for the genome assembly is JAUZMZ000000000 and the accession number of the 16S rRNA gene sequence is OR578921. The type strain, CC-R104^T^ (=LMG 33233^T^=UCCCB 171^T^), was isolated from the tissues of the red macroalgae *Chondrus crispus* collected on the northern Portuguese coast.

## supplementary material

10.1099/ijsem.0.006483 Uncited Supplementary Material 1.

## Abbreviations

AIA: actinomycete isolation agar
ANI: average nucleotide identity
BGC: biosynthetic gene cluster
CDS: coding sequence
CZA: Czapek-dox agar
dDDH: digital DNA-DNA hybridization
Dpm: 2,6-diaminopimelic acid
DSMZ: German collection of microorganisms and cell cultures
FAME: fatty acid methyl ester
G+C: guanine plus cytosine
GGDC: genome-to-genome distance calculator
GYM: glycerol yeast malt
HPLC: high-performance liquid chromatography
ISP: International Streptomyces project
JCM: Japan collection of microorganisms
MA: marine agar
Mb: megabyte
MK: menaquinone
ML: maximum-likelihood
NAPAA: non-alpha poly-amino acids
NJ: neighbor joining
NPS: nutrient-poor sediment extract
NRP: non-ribosomal peptide
PDA: potato-dextrose agar
RiPP: ribosomally synthesized and post-translationally modified peptide
TLC: thin-layer chromatography
TSA: tryptic soy agar
TSB: tryptic soy broth
