# Supplementary material for: Nocardiopsis codii sp. nov., and Rhodococcus chondri sp. nov., two novel actinomycetal species isolated from macroalgae collected in the northern Portuguese coast
Source: Int J Syst Evol Microbiol. 2024 Sep 10;74(9):006483. doi: 10.1099/ijsem.0.006483 (PMC11475406; doi:10.1099/ijsem.0.006483)
Supplement: Uncited Supplementary Material 1. [file ijsem-74-06483-s001.pdf]

Nocardiopsis codii sp. nov., *and Rhodococcus chondri* sp. nov., two novel Actinomycetal species isolated from macroalgae collected in the northern Portuguese coast

Mariana Girão[1,2], Zoé Lequint[1,3], Adriana Rego[1,] Isabel Costa[1], Diogo Neves Proença[4], Paula V. Morais[4], Maria F. Carvalho[1,2]

[1] CIIMAR - Interdisciplinary Centre of Marine and Environmental Research, University of Porto, Portugal
[2] ICBAS - School of Medicine and Biomedical Sciences, University of Porto, Portugal
[3] Polytech Clermont, University Clermont Auvergne, France
[4] University of Coimbra, Department of Life Sciences, CEMMPRE, ARISE, Coimbra, Portugal

***Correspondence**:
Mariana Girão - mariana.martins@ciimar.up.pt

Supplementary Material

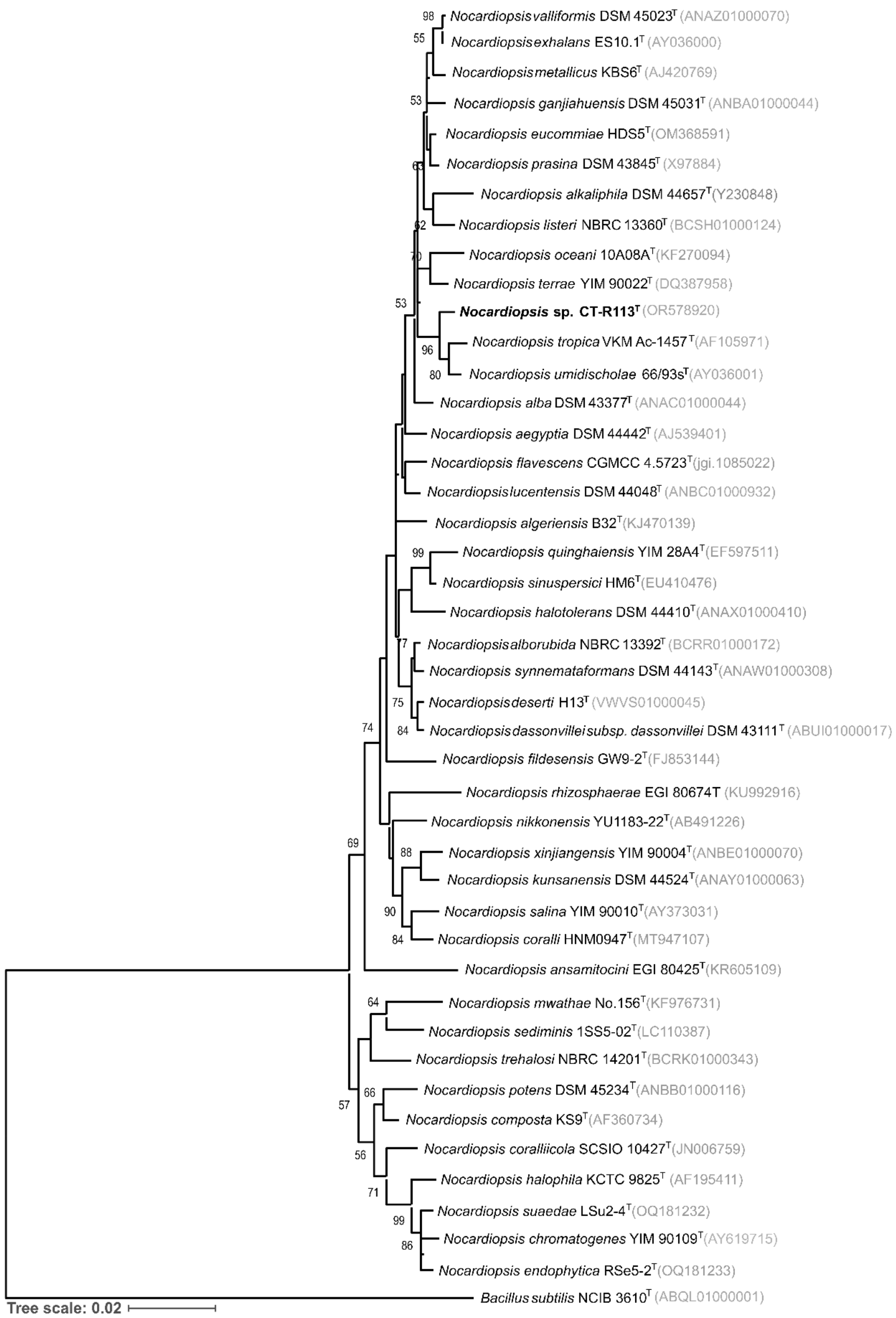

Fig. S1. NJ phylogenetic tree based on 16S rRNA gene sequences (1,551 nt), showing the relationship between strain CT-R113^T and the available type strains within the genus *Nocardiopsis*. Accession numbers are indicated in brackets. Values at the nodes indicate bootstrap values of 50% and above, obtained based on 1,000 resampling events. *Bacillus subtilis* NCIB 3610^T was used as outgroup. Scale bar, 2 inferred nucleotide substitution per 100 nucleotides.

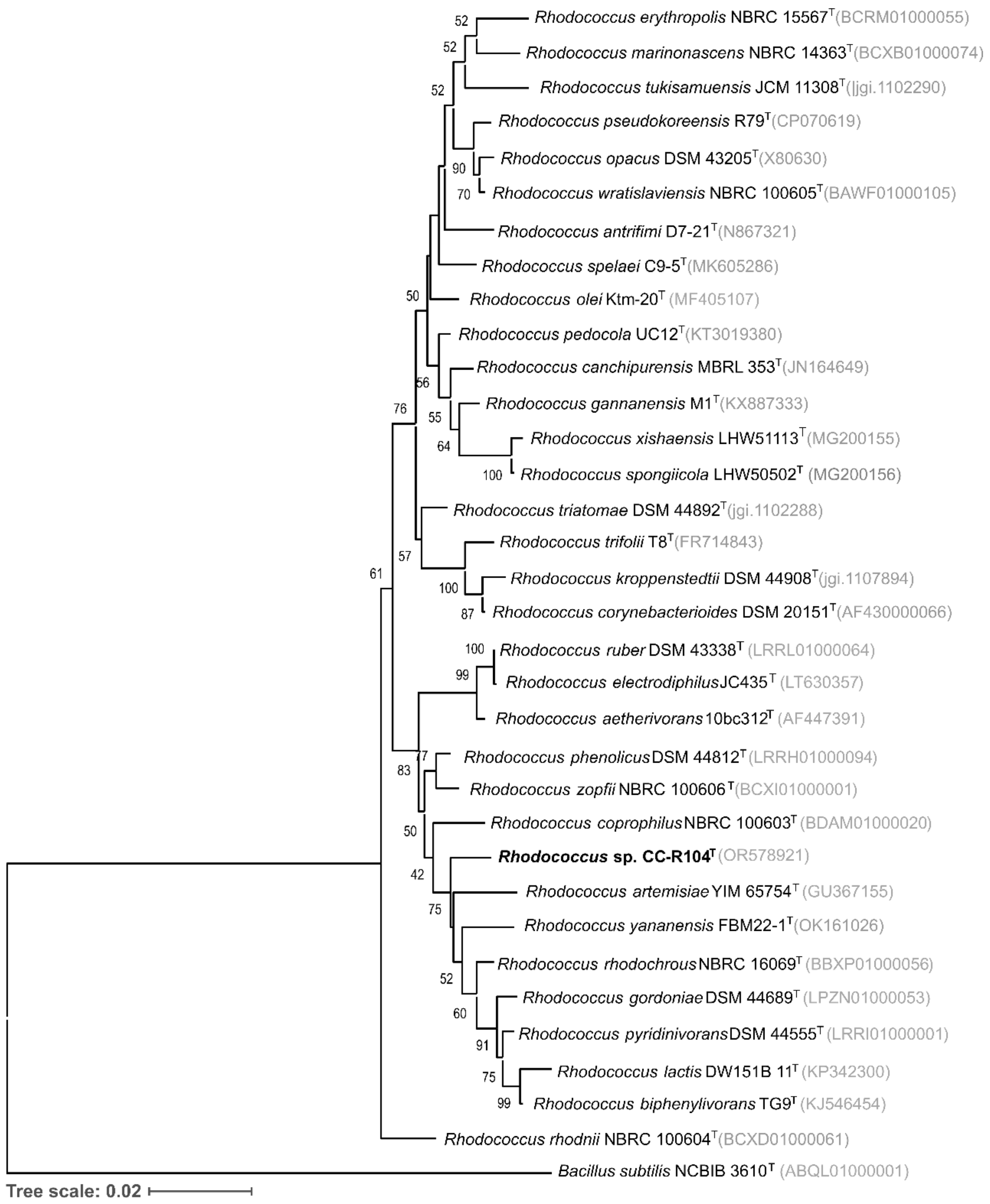

Fig. S2. NJ phylogenetic tree based on 16S rRNA gene sequences (1,553 nt), showing the relationship between strain CC-R104^{T} and the available type strains within the genus *Rhodococcus*. Accession numbers are indicated in brackets. Values at the nodes indicate bootstrap values of 50% and above, obtained based on 1,000 resampling events. *Bacillus subtilis* NCIB 3610^{T} was used as outgroup. Scale bar, 2 inferred nucleotide substitution per 100 nucleotides.

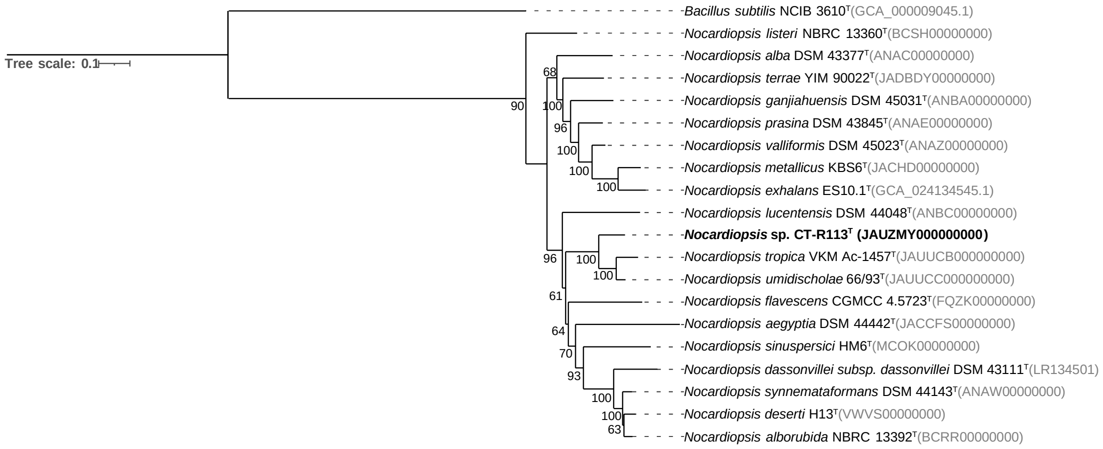

Fig. S3. NJ phylogenomic tree based on 400 universal marker genes, showing the relationship between strain CT-R113^{T} and the closest related type strains within the genus *Nocardiopsis*. Accession numbers are indicated in brackets. Values at the nodes indicate bootstrap values of 50% and above obtained based on 1,000 resampling events. *Bacillus subtilis* NCIB 3610^{T} was used as outgroup. Scale bar, 10 inferred nucleotide substitution per 100 nucleotides.

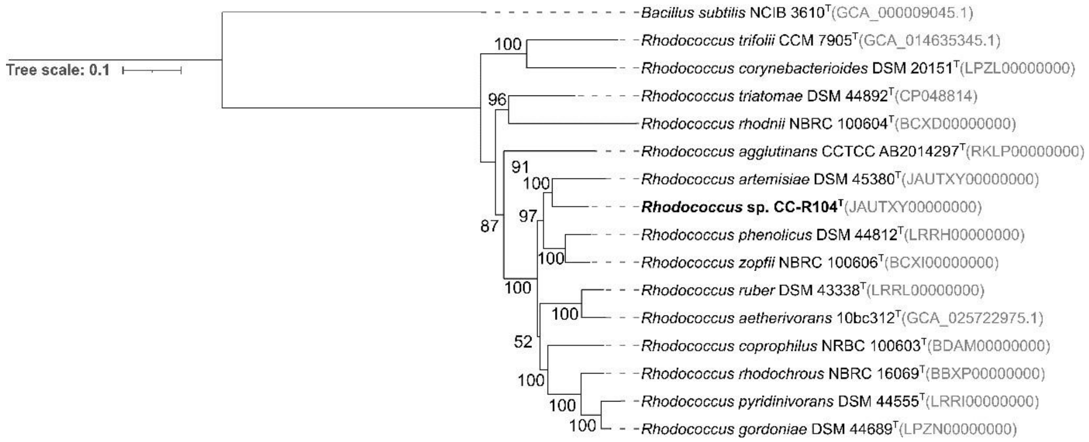

Fig. S4. NJ phylogenomic tree based on 400 universal marker genes, showing the relationship between strain CC-R103^{T} and the closest related type strains within the genus *Rhodococcus*. Accession numbers are indicated in brackets. Values at the nodes indicate bootstrap values of 50% and above obtained based on 1,000 resampling events. *Bacillus subtilis* NCIB 3610^{T} was used as outgroup. Scale bar, 10 inferred nucleotide substitution per 100 nucleotides.

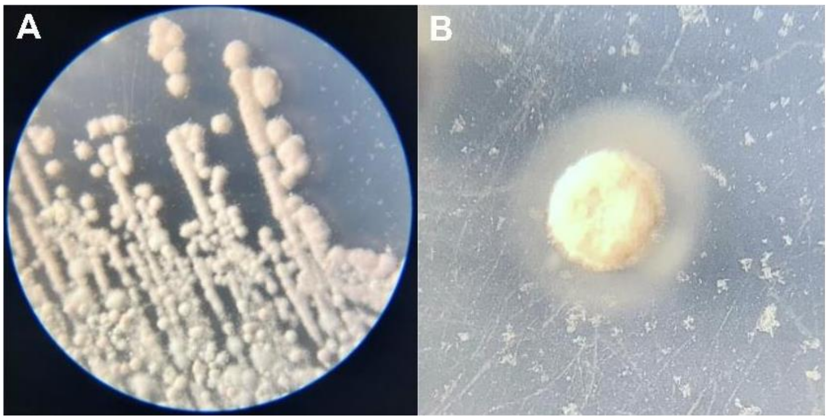

Fig. S5. Morphology of strain CT-R113^T colonies oin TSA medium, observed using a binocular magnifier (Leica ZOOM 2000) with magnifications of (A) 10x and (B) 30x.

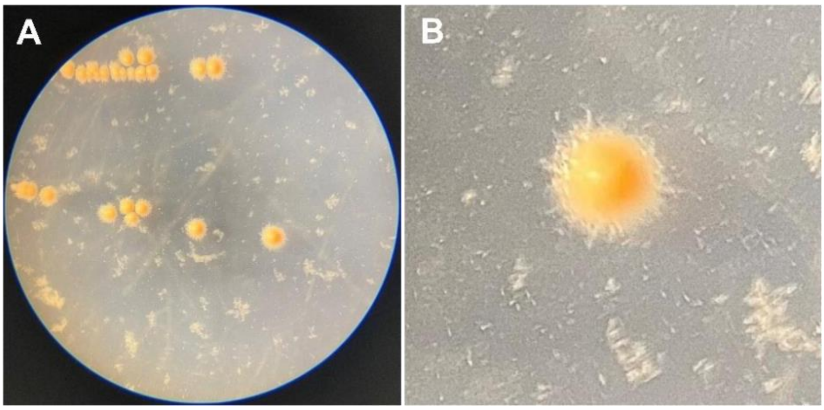

Fig. S6. Morphology of colonies of strain CC-R104^T on TSA medium, observed using a binocular magnifier (Leica ZOOM 2000) with magnifications of (A) 10x and (B) 30x.

Table S1. ANI (%) between the genome of strain CT-R113^{T} and the closest related type strains within the genus *Nocardiopsis*. Query genome: *Nocardiopsis* sp. CT-R113^{T} (JAUZMY000000000).

**Reference Genome**	**ANI (%)**
Nocardiopsis umidischolae 66/93^{T} (JAUUCC000000000)	90.6
Nocardiopsis tropica VKM Ac-1457^{T} (JAUUCB000000000)	89.9
Nocardiopsis synnemataformans DSM 44143^{T} (ANAW00000000)	85.6
Nocardiopsis dassonvillei subsp. dassonvillei DSM 43111^{T} (LR134501)	85.5
Nocardiopsis sinuspersici HM6^{T} (MCOK00000000)	85.5
Nocardiopsis deserti H13^{T} (VWVS00000000)	85.5
Nocardiopsis alborubida NBRC 13392^{T} (BCRR00000000)	85.4
Nocardiopsis terrae YIM 90022^{T} (JADBDY00000000)	83.4
Nocardiopsis ganjiahuensis DSM 45031^{T} (ANBA00000000)	83.3
Nocardiopsis aegyptia DSM 44442^{T} (JACCFS00000000)	83.3
Nocardiopsis metallicus KBS6^{T} (JACHD00000000)	83.3
Nocardiopsis exhalans ES10.1^{T} (GCA_024134545.1)	83.2
Nocardiopsis flavescens CGMCC 4.5723^{T} (FQZK00000000)	83.2
Nocardiopsis valliformis DSM 45023^{T} (ANAZ00000000)	83.0
Nocardiopsis lucentensis DSM 44048^{T} (ANBC00000000)	83.1
Nocardiopsis alba DSM 43377^{T} (ANAC00000000)	82.5
Nocardiopsis listeri NBRC 13360^{T} (BCSH00000000)	82.4

Table S2. ANI (%) between the genome of strain CC-R104^{T} and the closest related type strains within the genus *Rhodococcus*. Query genome: *Rhodococcus* sp. CC-R104^{T} (JAUTXY000000000).

**Reference Genome**	**ANI (%)**
Rhodococcus rhodochrous NBRC 16069^{T} (BBXP00000000)	81.7
Rhodococcus gordoniae DSM 44689^{T} (LPZN00000000)	81.6
Rhodococcus pyridinivorans DSM 44555^{T} (LRRI00000000)	81.5
Rhodococcus phenolicus DSM 44812^{T} (LRRH00000000)	81.4
Rhodococcus zopfii NBRC 100606^{T} (BCXI00000000)	81.3
Rhodococcus coprophilus NBRC 100603^{T} (BDAM00000000)	81.2
Rhodococcus artemisiae YIM 65754^{T} (JAUTXY000000000)	81.2
Rhodococcus aetherivorans 10bc312^{T} (GCA_025722975.1)	81.0
Rhodococcus ruber DSM 43338^{T} (LRRL00000000)	80.9
Rhodococcus agglutinans CCTCC AB2014297^{T} (RKLP00000000)	79.6
Rhodococcus triatomae DSM 44892^{T} (CP048814)	78.8
Rhodococcus rhodnii NBRC 100604^{T} (BCXD00000000)	78.6
Rhodococcus corynebacterioides DSM 20151^{T} (LPZL00000000)	78.3
Rhodococcus trifolii T8^{T} (GCA_014635345.1)	77.9

Table S3. Secondary metabolite biosynthetic gene clusters (BGCs) identified by antiSMASH (version 7.1.0) in the genomes of strains CT-R113^{T}, JCM 10877^{T} and JCM 11758^{T}. Relaxed detection settings and all extra features were selected.

CT-R113^{T}

Region	Type of compound	Most similar known cluster	Similarity (%)	MiBiG accession	BGC origin	Length (nt)
1.1	T1PKS	Aldgamycin J	13	BGC0001396	*Streptomyces* sp. A1(2016)	323,681 – 381,248
2.1	Ectoine	Ectoine	75	BGC0002052	*Streptomyces* sp.	22,28 – 32,681
2.2	T2PKS	Formicamycins A-M	9	BGC0001590	*Streptomyces* sp. KY5	350,126 – 422,602
4.1	Butyrolactone	Murayaquinone	3	BGC0001675	*Streptomyces griseoruber*	7,683 – 18,786
4.2	NI-siderophore	Desferrioxamine E	100	BGC0001478	*Streptomyces* sp. ID38640	154,004 – 183,926
5.1	Nucleoside	Huimycin	70	BGC0002354	*Kutzneria albida* DSM 43870	217,701 – 238,063
6.1	Butyrolactone	-	-	-	-	123,541 – 134,584
7.1	NRPS	Incednine	4	BGC0000078	Streptomyces sp. ML694-90F	21,12 – 65,568
8.1	NRPS	Surugamide A	19	BGC0001792	*Streptomyces albidoflavus*	64,853 – 124,582
11.1	Terpene	Isorenieratene	87	BGC0001456	*Streptomyces argillaceus*	128,703 – 154,316
15.1	Lassopeptide/T3PKS	-	-	-	-	13,241 – 73,434
15.2	Terpene	Legonindolizidine A6	12	BGC0002666	*Streptomyces* sp. MA37	73,708 – 95,084
15.3	RiPP-like	-	-	-	-	102,276 -112,572
16.1	Lanthipeptide-class-i	-	-	-	-	93,18 – 118,636
16.2	Guanidinotides	Actagardine	6	BGC0000495	*Actinoplanes garbadinensi*	159,992 – 173,472
19.1	HR-T2PKS/Butyrolactone	Colabomycin E	13	BGC0000213	*Streptomyces aureus*	1 – 39,94
19.2	Ectoine	Kosinostatin	4	BGC0001073	*Micromonospora* sp. TP-A046	143,778 – 149,237
22.1	Lanthipeptide-class-iv	Duramycin	25	BGC0001579	*Streptomyces cinnamoneus*	61,069 – 83,645
24.1	NRP-metallophore/NRPS/T1PKS	Coelibactin	90	BGC0000324	*Streptomyces coelicolor* A3(2	41,121 – 120,809
43.1	NI-siderophore	Nonactin	33	BGC0000244	*Streptomyces griseus* subsp. *griseus*	21,682 – 45,602
48.1	T3PKS	Feglymycin	26	BGC0001233	*Streptomyces* sp. DSM 11171	1 – 22,133
57.1	Oligosaccharide/Other,PKS-like	Kosinostatin	6	BGC0001073	*Micromonospora* sp. TP-A0468	1 – 27,728
61.1	Lanthipeptide-class-iii	-	-	-	-	5,123 – 20,015
65.1	RRE-containing	-	-	-	-	1 – 11,785
67.1	Thiopeptide,LAP	-	-	-	-	1 – 7,838
68.1	NRPS	-	-	-	-	1 – 5,932
75.1	NRPS	-	-	-	-	1 – 2,515

JCM 10877^T

Region	Type of compound	Most similar known cluster	Similarity (%)	MiBiG accession	BGC origin	Length (nt)
16.1	Lassopeptide	Streptomonomicin	40	BGC0001176	*Streptomonospora alba*	1 – 11,342
25.1	Terpene	Isorenieratene	18	BGC0001227	*Streptomyces collinus* Tu 365	1 – 10,074
38.1	RiPP-like	Funisamine	7	BGC0001944	*Streptosporangium* sp.	1 – 9,077
60.1	Lanthipeptide-class-i	-	-	-	-	1 – 7,321
130.1	Terpene	-	-	-	-	1 – 5,673
153.1	Other	Rubradirin	6	BGC0000141	*Streptomyces achromogenes* subsp. *rubradiris*	1 – 5,431
212.1	NI-siderophore	Desferrioxamine E	100	BGC0001478	*Streptomyces* sp. ID38640	1 – 4,767
281.1	RRE-containing	-	-	-	-	1 – 4,316
332.1	Ectoine	Ectoine	75	BGC0002052	*Streptomyces* sp.	1 – 4,048
383.1	NRPS	-	-	-	-	1 – 3,766
447.1	Butyrolactone	-	-	-	-	1 – 3,562
455.1	T1PKS	Nanchangmycin	30	BGC0000105	*Streptomyces nanchangensis*	1 – 3,536
475.1	Butyrolactone	-	-	-	-	1 – 3,436
615.1	Terpene	Isorenieratene	37	BGC0001456	*Streptomyces argillaceus*	1 – 2,956
712.1	NRPS-like	-	-	-	-	1 – 2,697
844.1	PKS-like	Calicheamicin	2	BGC0000033	*Micromonospora echinospora*	1 – 2,415
852.1	T1PKS	-	-	-	-	1 – 2,405

JCM 11758^T

Region	Type of compound	Most similar known cluster	Similarity (%)	MiBiG accession	BGC origin	Length (nt)
1.1	T3PKS	-	-	-	-	27,844 – 68,902
1.2	Terpene/Lassopeptide	Legonindolizidine A6	12	BGC0002666	*Streptomyces* sp. MA37	132,834 – 175,451
1.3	RiPP-like	-	-	-	-	211,985 – 222,281
2.1	T2PKS/Furan	Accramycin A	10	BGC0002315	*Streptomyces* sp.	93,728 – 166,205
3.1	NI-siderophore	Petrichorin A	11	BGC0002315	*Streptomyces* sp.	23,524 – 53,452
3.2	Betalactone	Formicamycins A-M	4	BGC0001590	*Streptomyces* sp. KY5	55,433 – 86,522
6.1	CDPS	-	-	-	-	72,627 – 93,475
7.1	T1PKS/NRPS-like/NRPS	a201a	10	BGC0001138	*Marinactinospora thermotolerans*	15,384 – 72,675
10.1	HR-T2PKS/Butyrolactone/Thioamide-NRP	Colabomycin E	13	BGC0000213	*Streptomyces aureus*	12,864 – 96,777
22.1	NRPS-like	Guanipiperazine A	80	BGC0002582	*Streptomyces chrestomyceticus*	39,858 – 78,026

24.1	NRPS	Malonomycin	44	BGC0001942	*Streptomyces rimosus* subsp. *paromomycinus*	47,621 – 77,767
36.1	Terpene	Isorenieratene	87	BGC0001456	Streptomyces argillaceus	1 – 18,804
38.1	Butyrolactone	SF2575	4	BGC0000269	Streptomyces sp. SF2575	55,38 – 61,42
40.1	Guanidinotides	Mannopeptimycin	7	BGC0000388	Streptomyces hygroscopicus	3,633 – 26,186
54.1	Oligosaccharide/Other/PKS-like	Mycinamicin II	14	BGC0000102	Micromonospora griseorubida	3,909 – 48,935
61.1	NRPS	CDA1b	12	BGC0000315	Streptomyces coelicolor A3(2)	1 – 43,978
65.1	NRPS	Malonomycin	27	BGC0001942	Streptomyces rimosus subsp. paromomycinus	7,602 – 42,375
86.1	Lanthipeptide-class-iii	Auroramycin	2	BGC0001522	Streptomyces filamentosus	7,621 – 29,216
90.1	Butyrolactone	-	-	-	-	19,237 – 30,18
92.1	T1PKS	Aldgamycin J	13	BGC0001396	Streptomyces sp. A1(2016)	1 – 28,851
95.1	NRPS	Coelibactin	45	BGC0000324	Streptomyces coelicolor A3(2)	1 – 26,759
101.1	Ectoine	-	-	-	-	20,351 – 25,868
107.1	T1PKS	Uncialamycin	12	BGC0001377	Streptomyces uncialis	1 – 23,592
112.1	NRP-metallophore,NRPS	Coelibactin	27	BGC0000324	Streptomyces coelicolor A3(2)	1 – 24,314
129.1	Lanthipeptide-class-iv	-	-	-	-	6,791 – 19,571
134.1	RiPP-like	-	-	-	-	12,437 – 18,779
137.1	Lanthipeptide-class-iii	-	-	-	-	1 – 13,356
145.1	NRPS	Incednine	4	BGC0000078	Streptomyces sp. ML694-90F3	1 – 17,092
153.1	NRPS	Acyldepsipeptide 1	15	BGC0001967	Streptomyces hawaiiensis	1 – 14,712
158.1	T1PKS/NRPS/NRPS-like	BE-43547A1	10	BGC0001330	Micromonospora sp. RV43	1 – 14,397
242.1	T1PKS	-	-	-	-	1 – 3,310

T1PKS: Type I polyketide synthase
T2PKS: Type 2 polyketide synthase
T3PKS: Type 3 polyketide synthase
NRPS: Non-ribosomal peptide synthetase
RiPP-like: Other unspecified ribosomally synthesised and post-translationally modified peptide product
NRPS-like: Non-ribosomal peptide synthetase-like fragment
PKS-like: Polyketide synthase-like fragment

Table S4. Secondary metabolite biosynthetic gene clusters (BGCs) identified by antiSMASH (version 7.1.0) in the genomes of strains CC-R104^{T} and DSM 45380^{T}. Relaxed detection settings and all extra features were selected.

CC-R104^{T}

Region	Type of compound	Most similar known cluster	Similarity (%)	MiBiG accession	BGC origin	Length (nt)
2.1	Terpene	5-dimethylallylindole-3-acetonitrile	55	BGC0002128	*Streptomyces coelicolor* A3(2)	20,325 - 41,314
18.1	Terpene	Isorenieratene	42	BGC0000664	*Streptomyces griseus subsp. griseus* NBRC 13350	8,206,573 - 8,214,963
25.1	Redox-cofactor	-	-	-	-	15,707 - 38,518
40.1	NRPS	-	-	-	-	1 - 34,048
49.1	Betalactone/NRPS-like	Hedamycin	6	BGC0000233	*Streptomyces griseoruber*	1 - 45,603
61.1	Terpene	SF2575	4	BGC0000269	*Streptomyces* sp. SF2575	1 - 53,273
106.1	NAPAA	Rifamorpholine A	3	BGC0001759	*Amycolatopsis* sp.	1 - 113,830
109.1	RiPP-like	-	-	-	-	5,328 – 16,131
161.1	Ectoine	Ectoine	75	BGC0000853	*Streptomyces anulatus*	1 – 3,366
162.1	NRPS	-	-	-	-	1 – 10,151
171.1	NRPS	Atratumycin	7	BGC0001975	*Streptomyces atratus*	1 – 9,747
175.1	NRPS	Tetrocarcin A	4	BGC0000162	*Micromonospora chalcea*	1 – 9,313
191.1	NRPS-like	-	-	-	-	1 – 8,416
194.1	T1PKS	-	-	-	-	1 – 8,250
207.1	NRPS-like	-	-	-	-	1 – 7,676
230.1	NRPS	-	-	-	-	1 – 5,946
279.1	NRPS	-	-	-	-	1 – 3,860
280.1	NRPS	-	-	-	-	1 – 3,809
308.1	NRPS	-	-	-	-	1 – 2,935
316.1	NRPS	-	-	-	-	1 – 2,724
325.1	NRPS-like	-	-	-	-	1 – 2,405
355.1	NRPS	-	-	-	-	1 – 1,485
363.1	NRPS-like	-	-	-	-	1 – 3,347
366.1	NRPS	-	-	-	-	1 – 3,335

DSM 45380ᵀ

Region	Type of compound	Most similar known cluster	Similarity (%)	MiBiG accession	BGC origin	Length (nt)
1.1	NRPS	-	-	-	-	1 – 24,688
1.2	Terpene	SF2575	6	BGC0000269	*Streptomyces* sp. SF2575	216,338 – 237,456
1.3	Ectoine	Ectoine	75	BGC0000853	*Streptomyces anulatus*	681,998 – 692,399
2.1	Butyrolactone	-	-	-	-	655,789 – 666,925
4.1	T1PKS	-	-	-	-	369.022 – 413.992
6.1	NAPAA	ε-Poly-L-lysine	100	BGC0002174	*Epichloe festucae*	1 – 3,894
7.1	NRPS/NRPS metallophore	Amychelin A	33	BGC0002544	*Amycolatopsis methanolica*	1 – 39,399
7.2	RiPP-like	-	-	-	-	295,872 – 306,675
8.1	Redox-cofactor	-	-	-	-	191,139 – 213,953
8.2	NRPS	Cinnapeptin	7	BGC0002108	*Streptomyces viridosporus* ATCC 14672	221,462 – 317,220
10.1	Terpene	Isorenieratene	37	BGC0000664	*Streptomyces griseus subsp. griseus* NBRC 13350	12,679 – 33,653
11.1	NRPS/T1PKS/Betalactone	Scopranone A	5	BGC0002558	*Streptomyces* sp.	74,137 – 148,225
12.1	NRPS	-	-	-	-	13,660 – 87,160
12.2	NRPS	-	-	-	-	139,749 – 198,594
18.1	NRPS/Betalactone	-	-	-	-	1 – 32,486
23.1	NRPS	Chloramphenicol	17	BGC0000893	*Streptomyces venezuelae* ATCC 10712	35,764 – 72,800
25.1	NRPS/T1PKS	-	-	-	-	1 – 43,905

NRPS: Non-ribosomal peptide synthetase
NRPS-like: Non-ribosomal peptide synthetase-like fragment
NAPAA: Non-alpha poly-amino acids like e-Polylysin
RiPP-like: Other unspecified ribosomally synthesised and post-translationally modified peptide product
T1PKS: Type I polyketide synthase
